# Mitochondrial Dysfunction in Advanced Liver Disease: Emerging Concepts

**DOI:** 10.3389/fmolb.2021.772174

**Published:** 2021-11-23

**Authors:** Ingrid W. Zhang, Cristina López-Vicario, Marta Duran-Güell, Joan Clària

**Affiliations:** ^1^ Biochemistry and Molecular Genetics Service, Hospital Clínic–IDIBAPS, Barcelona, Spain; ^2^ European Foundation for the Study of Chronic Liver Failure (EF Clif) and Grifols Chair, Barcelona, Spain; ^3^ CIBERehd, Barcelona, Spain; ^4^ Department of Biomedical Sciences, University of Barcelona, Barcelona, Spain

**Keywords:** mitochondrial dysfunction, systemic inflammation, organ failure, cirrhosis, acute decompensation, immunometabolism

## Abstract

Mitochondria are entrusted with the challenging task of providing energy through the generation of ATP, the universal cellular currency, thereby being highly flexible to different acute and chronic nutrient demands of the cell. The fact that mitochondrial diseases (genetic disorders caused by mutations in the nuclear or mitochondrial genome) manifest through a remarkable clinical variation of symptoms in affected individuals underlines the far-reaching implications of mitochondrial dysfunction. The study of mitochondrial function in genetic or non-genetic diseases therefore requires a multi-angled approach. Taking into account that the liver is among the organs richest in mitochondria, it stands to reason that in the process of unravelling the pathogenesis of liver-related diseases, researchers give special focus to characterizing mitochondrial function. However, mitochondrial dysfunction is not a uniformly defined term. It can refer to a decline in energy production, increase in reactive oxygen species and so forth. Therefore, any study on mitochondrial dysfunction first needs to define the dysfunction to be investigated. Here, we review the alterations of mitochondrial function in liver cirrhosis with emphasis on acutely decompensated liver cirrhosis and acute-on-chronic liver failure (ACLF), the latter being a form of acute decompensation characterized by a generalized state of systemic hyperinflammation/immunosuppression and high mortality rate. The studies that we discuss were either carried out in liver tissue itself of these patients, or in circulating leukocytes, whose mitochondrial alterations might reflect tissue and organ mitochondrial dysfunction. In addition, we present different methodological approaches that can be of utility to address the diverse aspects of hepatocyte and leukocyte mitochondrial function in liver disease. They include assays to measure metabolic fluxes using the comparatively novel Biolog’s MitoPlates in a 96-well format as well as assessment of mitochondrial respiration by high-resolution respirometry using Oroboros’ O2k-technology and Agilent Seahorse XF technology.

## 1 Introduction

Although mitochondrial damage has beneficial effects in response to acute injuries or infections through promotion of pro-inflammatory responses, it might be deleterious in diseases associated with chronic inflammation such as advanced liver cirrhosis (Mansouri et al., 2018). Therapeutic interventions aimed at restoring the deranged intermediate metabolism in immune cells might sever the links between excessive mitochondrial damage and deranged immune response and have beneficial effects in patients with acute decompensation (AD) of cirrhosis and acute-on-chronic liver failure (ACLF), a syndrome developing in patients with AD characterized by the presence of organ failure(s).

AD cirrhosis is defined by acute development of ascites, hepatic encephalopathy, gastrointestinal haemorrhage or bacterial infection, or any combination of these, and ACLF is a syndrome with a prevalence of 30% in patients hospitalized for AD cirrhosis (Moreau et al., 2013). An evidence-based definition of ACLF was introduced for the first time in 2013, as a result of the prospective and European-wide CANONIC study (Moreau et al., 2013). The short-term mortality rate of patients with ACLF is 15 times higher than that of patients with mere AD, and the main cause of death is multiple organ failure. The current European and American definitions are based on the presence of organ failures, and the severity of the disease (grade I-III) is determined according to the number of organ failures (Moreau et al., 2013). On the pathophysiological level, ACLF is characterized by the co-existence of systemic inflammation and immunoparesis, thereby rendering these patients more susceptible to bacterial infections (Bernsmeier et al., 2015; Clària et al., 2016; Fernández et al., 2017). ACLF shares pathophysiological similarities with sepsis, during which immune cells undergo metabolic reprogramming to fuel the hyper-inflammatory state. Therefore, it can be assumed that mitochondria, which were recently dubbed as the powerhouses of immunity (Mills et al., 2017), play a crucial role in both conditions. Whereas the evidence is overwhelming and convincing in sepsis, the role of mitochondria in ACLF development due to its relatively recent definition is less well described.

The intricate link between mitochondrial dysfunction and subsequent disturbances in metabolic pathways, leading to shortage in adenosine triphosphate (ATP), excessive storage of fat and leakage of reactive oxygen species (ROS) is well acknowledged (Ott et al., 2007; Aon et al., 2014). In recent years, however, the crosstalk between mitochondria and the immune system has attracted increased attention, to the point that one entire research field termed immunometabolism is dedicated to disentangle this extremely complex network. Recent studies helped to increase the recognition that the metabolic-immunoregulatory crosstalk is bidirectional. On the one hand, immune cells have to transition from a metabolic quiescent to an active state during immune response, and on the other hand, altered metabolism controls immune cell differentiation, function and fate (Angajala et al., 2018). More specifically, mitochondrial intermediate metabolism affects cytokine production, i.e. M1 macrophage polarization is supported by a discontinuous tricarboxylic acid (TCA) cycle, whereas mitochondrial β-oxidation is required for M2 macrophage polarization (Tannahill et al., 2013; Huang et al., 2014; Jha et al., 2015). Additionally, mitochondria are able to activate different intracellular signaling pathways necessary to defend the host against pathogens such as the mitochondrial anti-viral signaling (MAVS) and activation of the nucleotide-binding oligomerization domain (NOD)-, leucine-rich repeat (LRR)- and pyrin domain-containing protein 3 (NLRP3) inflammasome by mitochondrial DNA (mtDNA) (Zhong et al., 2018). Apart from mtDNA, mitochondria also release proteins, lipids, metabolites and ROS. Mitochondria are the major contributor to ROS production in most mammalian cells, and ROS is implicated in redox signaling from mitochondria to other organelles of the cell. The molecules released by mitochondria serve as damage-associated molecular patterns (DAMPs). DAMPs interact with pattern-recognition receptors (PRRs) expressed mainly by cells of the innate immune system in an autocrine, paracrine or endocrine manner (Vénéreau et al., 2015; Miliotis et al., 2019). Since mitochondria are the organelles where metabolic pathways converge, they are also able to reshape the innate and adaptive immune responses by modulating the microenvironment. For instance, a lipid-rich tumor microenvironment favours expansion of the pro-tumoral, interleukin (IL)-17 secreting γδ T cell subset over the anti-tumoral interferon (IFN)γ producing γδ T cells (Lopes et al., 2021), and since glycolysis controls the translation of IFNγ-mRNA, the lack of glucose impairs T cell cytokine production (Chang et al., 2013). Another example is that oligomycin can block the expression of early activation markers after T-cell receptor ligation and blunt subsequent T cell proliferation (Chang et al., 2013).

Mitochondria do not merely release immunostimulatory molecules, but also represent intracellular sites of inflammatory signaling. For example, among the 22 nucleotide-binding leucine-rich repeat receptors or NOD-like receptors (NLRs), NOD5 (or NLRX1) has a predicted mitochondrial import signal (Tattoli et al., 2008). In addition, NLRP3, after being stimulated with monosodium urate, alum or nigericin, relocates from cytoplasmic structures and the ER to mitochondria-associated ER membranes (MAM) (Zhou et al., 2011). Interestingly, NLRP3 has the potential to drive fibrogenesis independent from inflammasome-regulated cytokines IL-1β/IL18 and processing of caspase 1. Instead, NLRP3 operates at mitochondria in fibroblasts and modulates their ROS production to augment profibrogenic pathways (Bracey et al., 2014).

In analogy to mitochondria being the metabolic hub of the cell, the liver can be seen as the metabolic hub of the body. Often, similar to the underestimation of the role of mitochondria in immune responses, the perception of the liver is reduced to its metabolic functions. This notion should be revised, since the liver is a site of complex immunological activity, involved in the production of acute phase proteins, coagulation and complement factors, cytokines and albumin, and contains a large population of resident immune cells. Innate lymphocytes in the liver comprise natural killer (NK) cells, NK T cells (NKT), mucosal associated invariant T cells and γδ T cells (Doherty et al., 1999; Kenna et al., 2003; Kenna et al., 2004; Dusseaux et al., 2011). Regarding cells of the adaptive immune system, the liver is particularly enriched in CD8^+^ T cells, activated T cells and memory T cells (Norris et al., 1998). Of note, hepatocytes express variable levels of class II MHC molecules and are capable of presenting antigens to classical T cells (Thomson and Knolle, 2010). The healthy adult liver maintains a basal cytokine level including expression of pro-inflammatory IL-2, IL-7, IL-12, IL-15 and IFNγ, and anti-inflammatory IL-10, IL-13 and transforming growth factor β (TGFβ) (Golden-Mason et al., 2004; Kelly et al., 2006). Worthy of note, the immunological microenvironment in the liver is subjected to direct influence by macronutrients transported to the liver through the portal vein.

Given that recurring commonalities of hepatic diseases include increased ROS and reactive nitrogen species, diminished β-oxidation, defective oxidative phosphorylation and enhanced lipogenesis, we propose that immunometabolic derangement and bioenergetics failure is the phenotype underlying advanced liver cirrhosis including ACLF, similar to sepsis (Cheng et al., 2016).

In the following, we highlight the characteristics of immunometabolism that goes astray in advanced stages of liver cirrhosis, the role of dysfunctional mitochondria in development of organ failures in patients with ACLF, and propose therapeutic strategies aimed at rewiring mitochondrial intermediate metabolism.

## 2 Immunometabolism in the Setting of Advanced Liver Disease: Role of the Tricarboxylic Acid Cycle

**FIGURE 1:**
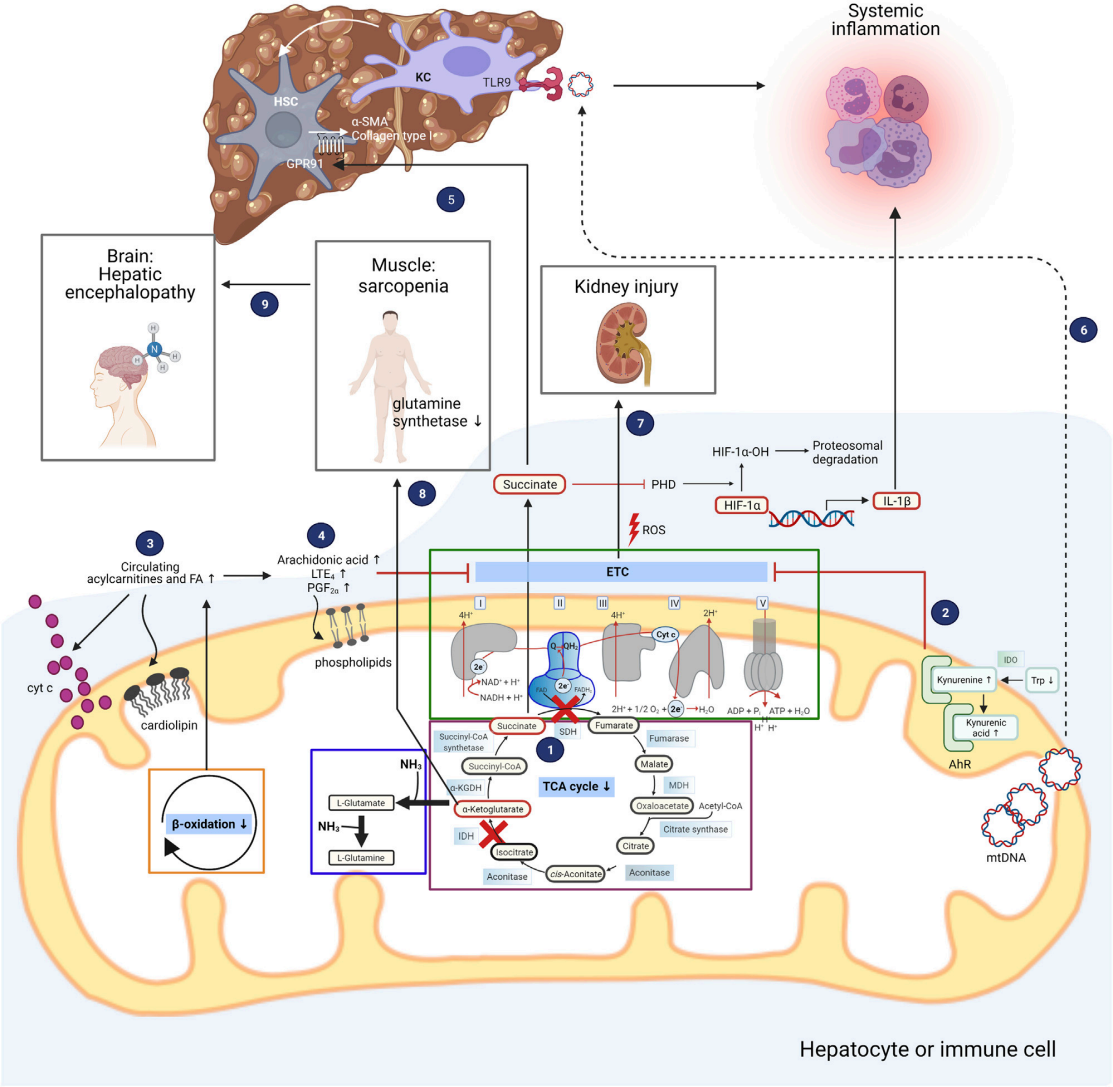
Deranged mitochondrial intermediate metabolism contributes to development of organ dysfunction in patients with acute decompensated liver cirrhosis. The different metabolic intermediate pathways located in mitochondria are depicted, and how their derangements contribute to the clinical symptoms of patients with acutely decompensated liver cirrhosis. The boxes inside the mitochondrion highlight the electron transport chain (ETC), the tricarboxylic acid (TCA) cycle, and glutamine synthesis as one possible pathway for ammonia detoxification. Peripheral mononuclear cells of patients with ACLF exhibit a discontinuous TCA cycle at the isocitrate and succinate dehydrogenase (IDH and SDH) level **(1)**. Succinate is transported into the cytosol where it inhibits prolyl hydroxylase (PHD), resulting in stabilization of hypoxia-inducible factor-1α (HIF-1α) and enhanced production of interleukin-1β (IL-1β). Untargeted metabolomics in serum of patients with AD cirrhosis and ACLF revealed increased degradation of tryptophan (Trp) through the kynurenine pathway with elevated kynurenine and kynurenic acid levels, which bind to aryl hydrocarbon receptor (AhR) localized in the intermembrane space of mitochondria **(2)**. Activation of AhR is implicated in attenuation of mitochondrial maximal respiration. Patients with AD cirrhosis and ACLF also exhibit elevated levels of fatty acids and acylcarnitines, pointing towards impaired β-oxidation **(3)**. Saturated fatty acids (FA) change the composition of cardiolipins which are enriched in the inner mitochondrial membrane, facilitating the release of cytochrome c. Targeted study of bioactive lipid mediators in patients with AD cirrhosis and ACLF revealed elevated levels of arachidonic acid and its products LTE_4_ and PGF_2α_ **(4)**. Arachidonic acid is able to modify the phospholipid composition of mitochondrial membranes, resulting in impaired function of the ETC. In addition to being involved in pro-inflammatory responses, succinate promotes fibrogenesis through its receptor GPR91 which is expressed on hepatic stellate cells (HSC), leading to upregulation of alpha-smooth muscle actin (α-SMA) and collagen type I **(5)**. Mitochondrial DNA (mtDNA) released from hepatocytes promote inflammation through binding to toll-like receptor 9 (TLR9) of liver resident Kupffer cells (KC) **(6)**, further contributing to fibrogenesis. Reactive oxygen species (ROS) generated by the electron transport chain and released by mitochondria promote cell death of kidney cells, eventually leading to kidney dysfunction and failure **(7)**. Hyperammonemia in patients with advanced liver cirrhosis leads to depletion of α-ketoglutarate in skeletal muscle and consequently lower TCA flux and ATP synthesis, translating into sarcopenia **(8)**. Along with muscle loss, significant amounts of glutamine synthetase activity is lost, promoting hepatic encephalopathy **(9)**. IDO, indoleamine-2,3-dioxygenase; MDH, malate dehydrogenase. Figure was created with BioRender.com.

Upon polarization of macrophages to the M1 phenotype, a discontinuity in the metabolic flux of the TCA cycle occurs at the isocitrate dehydrogenase (IDH) and succinate dehydrogenase (SDH) levels. As a consequence, succinate, a paradigmatic pro-inflammatory metabolite, is released into the cytosol, where it stabilizes the transcription factor hypoxia-inducible factor-1α (HIF-1α) through inhibition of prolyl hydroxylase (PHD) activity and promotes the production of ROS and IL-1β (Tannahill et al., 2013). In contrast, α-ketoglutarate (α-KG), another TCA cycle intermediate which functions as a branch point between glutamine metabolism and the TCA cycle, attenuates pro-inflammatory responses in M1 macrophages by suppressing nuclear factor-κB (NF-κB) pathway through PHD-dependent proline hydroxylation of IkappaB kinase (IKK) β (Liu et al., 2017). Therefore, M1 macrophage activation is strengthened by a low α-ketoglutarate/succinate ratio (Liu et al., 2017). Recently, we could demonstrate that the break point at SDH also exists in peripheral mononuclear cells of patients with ACLF (**1** in Figure 1), probably as a consequence of systemic inflammation in these patients (Zhang et al., 2021). This finding also generates the hypothesis that the break point serves to perpetuate the inflammatory responses through production of cytokines, which is promoted by the pro-inflammatory actions of succinate.

Mitochondria are regarded as the major cellular source of ROS production, but they can also be the target of excessive ROS generation. Mitochondrial ROS can damage metabolic enzymes such as α-ketoglutarate dehydrogenase (α-KGDH) and pyruvate dehydrogenase (Tretter and Adam-Vizi, 2005). α-KGDH, together with citrate synthase and IDH determine the overall rate of the TCA cycle (Cooney et al., 1981; Moreno-Sánchez et al., 1990), and α-KGDH is thought to be the key enzyme which limits the generation of NADH under exposure to oxidative stress (Tretter and Adam-Vizi, 2005). Patients with ACLF exhibit markedly elevated oxidative stress levels as represented by increased human nonmercaptalbumin 1 (HNA1), an oxidized form of albumin (Alcaraz-Quiles et al., 2018). Therefore, it is conceivable that in these patients, when α-KGDH is inhibited under high degree of oxidative stress, the supply of NADH to complex I of the electron transport chain (ETC) becomes limited, thus decreasing mitochondrial respiration and ATP production.

## 3 Amino Acid Metabolism in Advanced Liver Cirrhosis

Systemic inflammation initiated by DAMPs and pathogen-associated molecular patterns most probably is the initiator of metabolic derangements and subsequent organ failures and high mortality in patients with ACLF (Clària et al., 2016; Bajaj et al., 2020; Moreau et al., 2020). Activation of the innate immune system requires the integrated coordination of the metabolism of glucose, nonessential amino acids (AAs) and one-carbon (1C) metabolism, the latter being compartmentalized in the cytosol, mitochondria and nucleus. In decompensated cirrhosis, metabolic alterations comprise intense proteolysis and lipolysis, probably through stimulation of the hypothalamic-pituitary-adrenal axis by glucocorticoids (Ganeshan et al., 2019; Kelly and Pearce, 2020), leading to severe skeletal muscle catabolism (Moreau et al., 2020; Zaccherini et al., 2021). This is clinically most relevant, as sarcopenia is associated with the development of ACLF (Praktiknjo et al., 2019). In physiological conditions, AA catabolism is essential for the conservation of nitrogen and for maintaining physiologic concentrations of AAs, which cannot be stored up in case of dietary excess. Their catabolism occurs in the liver except for branched-chain amino acids, which are mainly metabolized by skeletal muscle and adipose tissue (Brosnan and Brosnan, 2006; Grohmann and Bronte, 2010). AA catabolism is used for controlling pathogen invasion and at the same time for regulating own immune responses (Bronte and Zanovello, 2005; Mellor and Munn, 2008). The metabolomics database of the landmark CANONIC study comprises 137 metabolites of which 43% are related to AAs. Reanalysis using weighted gene co-expression network analysis (WGCNA) identified 9 modules of co-regulated metabolites (Zaccherini et al., 2021). Worthy of note was the parallel increase of metabolite modules with markers of inflammation such as C-reactive protein (CRP), soluble CD163 (sCD163), sCD206, IL-6, tumor necrosis factor α (TNFα) and IL-10, indicating that inflammatory responses in patients with ACLF is accompanied by changes in AA metabolism (Zaccherini et al., 2021). The same study also suggested that in these patients, production of glutathione via the transsulfuration pathway and methionine renewal through the methionine salvage pathway are prioritized, probably to counteract systemic oxidative stress and to fuel the purine salvage pathway. In addition, N-formyl-L-methionine was reported to be increased in ACLF, suggesting enhanced conversion of serine to glycine and formate production in the folate cycle which forms part of the mitochondrial 1C metabolism.

In addition to reflecting disturbed AA metabolism, certain AAs and their derivatives exert biological functions, with the potential to impair organ functions (Clària et al., 2019). Patients with AD and ACLF present with reduced trytophan (Trp) levels, consistent with increased Trp degradation through the kynurenine pathway, giving rise to kynurenine, kynurenic and quinolinic acids (**2** in Figure 1). The first and rate-limiting step of the kynurenine pathway is catalyzed by the enzymes indoleamine-2,3-dioxygenase-1 (IDO-1) and tryptophan-2,3-dioxygenase (TDO) (Clària et al., 2019). *TDO2*, the gene encoding TDO, is mainly expressed in the liver, and IDO is known to be an important regulator of the immune system (Curti et al., 2009). In patients with ACLF, mRNA expression of *IDO1* and *IDO2* in peripheral blood mononuclear cells was increased, which could be related to the elevated plasma IFNγ levels in these patients (Clària et al., 2016; Clària et al., 2019). Increased expression of IDO depletes Trp in T cells, increasing uncharged tRNA levels, upon which the ribosomal stress-response kinase general control non-derepressible 2 (GCN2) is activated, leading to T cell anergy and proliferative arrest (Munn et al., 2005). Kynurenine and kynurenic acid exert immunomodulatory actions by binding to aryl hydrocarbon receptor (AhR) and G protein-coupled receptor 35 (GPR35) on immune cells (Wang et al., 2006; DiNatale et al., 2010). A portion of the AhR pool is localized in the intermembrane space of mitochondria, and ligand-induced activation of AhR attenuated mitochondrial maximal respiration (**2** in Figure 1) (Hwang et al., 2016). Increased kynurenine favours immune tolerance by inhibiting proliferation of T and NK cells and increasing proliferation of regulatory T cells and myeloid-derived suppressor cells (MDSCs), which might explain the positive association between higher baseline kynurenine pathway activity and the development of nosocomial infections and mortality in patients with AD cirrhosis (Clària et al., 2019). This finding further exemplifies the intricate link between metabolism and the immune system.

Pleiotropic effects on metabolic and immune processes were also described for glutamine and arginine, which are both inducers of mTORC1 (Chantranupong et al., 2016; Meng et al., 2020). Arginine functions as a precursor for metabolites with immunomodulatory properties such as polyamines and nitric oxide (NO) (Grohmann and Bronte, 2010) and is among the metabolites increased in patients with ACLF (Zaccherini et al., 2021). Increase in extracellular arginine increases oxygen consumption and mitochondrial spare respiratory capacity of T cells, indicating that arginine skews metabolism of activated T cells from glycolysis towards mitochondrial oxidative phosphorylation (OXPHOS), possibly through upregulation of serine biosynthesis which fuels the TCA cycle (Possemato et al., 2011; Geiger et al., 2016). Increase of intracellular arginine levels either through supplementation in medium or by inhibiting arginase (which metabolizes L-arginine to L-ornithine) resulted in less IFN-γ secretion and increased survival of antigen-activated T cells *in vitro* and *in vivo* (Geiger et al., 2016). Interestingly, patients with cirrhosis who developed poor outcome had higher levels of the arginine metabolites asymmetric dimethylarginine (ADMA) and symmetric dimethylarginine (SDMA) which adversely impact vascular reactivity and brain function (Bajaj et al., 2013; Bajaj et al., 2020).

## 4 Changes in Plasma and Mitochondrial Membrane Lipid Composition in Patients With Cirrhosis—Interplay Between Lipid Mediators and Mitochondrial Dysfunction

Inflammation is often associated with disturbances in cholesterol, fatty acid and phospholipid metabolism. This is also the case for patients with ACLF, who typically present with very low levels of high density lipoprotein (HDL) particles, probably attributed to the downregulation of reverse cholesterol transport mediated by acute phase responses (Feingold and Grunfeld, 2010; Trieb et al., 2020). It was proposed that the innate immune system differentially modifies macrophage cholesterol homeostasis in order to amplify the inflammatory response by activating the inflammasome and then to turn it off (Tall and Yvan-Charvet, 2015).

Untargeted blood lipidomics of cirrhotic patients with AD identified a specific lipid signature characterized by a generalized suppression of lipid species including the total polyunsaturated fatty acid (PUFA) pool and phospholipid families (i.e., lysophosphatidylcholine (LPC) and sphingomyelins), probably reflecting decreased hepatic biosynthetic capacity (López-Vicario et al., 2020; Clària et al., 2021). More specifically, LPC containing omega-3 PUFAs ranked among the lipids with greatest reduction in patients with AD and ACLF from the CANONIC cohort. Importantly, the decrease in LPC paralleled disease severity in patients with cirrhosis (Clària et al., 2021). Metabolomic analysis in a multicentre North American cohort complemented these findings, further demonstrating that patients who developed ACLF had lower serum phospholipids including phosphotidylethanolamine (PE), which is enriched in mitochondrial membranes, and higher estrone-3-sulfate level (Bajaj et al., 2020). An exception are fatty acids, which are notably increased due to enhanced lipolysis as indicated by blood accumulation of 2-hydroxyhexadecanoate, a by-product of lipolysis (Moreau et al., 2020; Zaccherini et al., 2021). At the same time, accumulation of acylcarnitines in the blood of patients indicates impaired translocation of fatty acids into mitochondrial matrix, the site of β-oxidation (**3** in Figure 1) (Moreau et al., 2020).

The alterations of blood lipid composition found in patients with decompensated cirrhosis are important, since it was shown that dietary fatty acids can remodel the lipid composition of mitochondrial membranes (Sullivan et al., 2018). Mitochondrial membranes are enriched with phospholipid species such as phosphatidylcholine (PC), PE, phosphatidylinositol (PI), phosphatidylserine (PS) and cardiolipin (Horvath and Daum, 2013; Sullivan et al., 2018). The latter is a unique structural lipid with four fatty acyl chains that favors mitochondrial membrane fusion and fission, cristae formation and respiratory function (Schlame and Ren, 2009; Paradies et al., 2014; Ren et al., 2014). The PUFA species incorporated into mitochondrial phospholipids are critical for optimal lipid molecular organization, lipid microenvironment and mitochondrial function including the respiratory enzymes involved in OXPHOS (Schlame and Ren, 2009; Genova and Lenaz, 2014; Paradies, 2014; Ruiz-Ramírez et al., 2015; Sullivan et al., 2018; Maekawa et al., 2019). Interestingly, studies using hepatic steatosis high calorie-fed animal models observed that increased hepatic availability of saturated FAs induced changes in the composition of mitochondrial membrane cardiolipins, leading to exacerbated cytochrome c released from the mitochondria to the cytosol and increased mitochondrial ROS generation (**3** in Figure 1) (Crescenzo et al., 2015; Ruiz-Ramírez et al., 2015). Furthermore, circulating saturated fatty acids are able to elicit a pro-inflammatory response, since they sensitize hepatocytes to TLR agonists (Csak et al., 2011).

The omega-6 PUFA linoleic acid (LA) is the major fatty acid in mitochondrial membranes and constitutes almost 90% of the cardiolipin fatty acyl chains, which bind with high affinity to respiratory chain enzymes and stabilize them (Planas-Iglesias et al., 2015; Sullivan et al., 2018). Moreover, the high amount of LA in cardiolipin has been positively correlated with cytochrome c oxidase activity (Fajardo et al., 2015). In an animal model of heart failure, LA has been shown to improve mitochondrial OXPHOS capacity in cultured rat cardiomyocytes (Maekawa et al., 2019). Albeit being important for mitochondrial membrane integrity, high amounts of LA directly reduced the mitochondrial marker enzyme citrate synthase, as well as routine and maximal mitochondrial respiration in a concentration-dependent manner (Shrestha et al., 2019). Furthermore, an excessive LA dietary intake leads to exacerbated arachidonic acid-derived lipid mediators, such as prostaglandin E_2_ (PGE_2_) and leukotriene B_4_ (LTB_4_) (Shrestha et al., 2019). In fact, this is clinically most relevant as patients with AD cirrhosis present with elevated levels of PGE_2_ which suppresses macrophage pro-inflammatory cytokine secretion and bacterial killing, mediated *via* the prostanoid type E receptor-2 (EP2) (O’Brien et al., 2014).

Since mitochondria play a critical role in the regulation of inflammation, bioactive lipid mediators generated under inflammatory conditions could play a paramount feature in mitochondrial function. Lipid mediators play a major role in the regulation of immune responses through generation of omega-6 PUFA-derived pro-inflammatory (i.e., PGs and LTs) and anti-inflammatory lipid mediators (specialized pro-resolving lipid mediators; SPM) derived from omega-3 PUFA (i.e., resolvins (Rv), maresins (MaR) and lipoxins (LX)), which are involved in the initiation and resolution of inflammation (Serhan, 2014; Dennis and Norris, 2015; López-Vicario et al., 2016). The main omega-3 PUFAs which serve as anti-inflammatory and pro-resolving lipid mediators or SPM precursors are docosahexaenoic acid (DHA) and eicosapentaenoic acid (EPA) (Serhan, 2014; López-Vicario et al., 2016). A targeted study of bioactive lipid mediators in patients with AD cirrhosis and ACLF further revealed higher circulating levels of arachidonic acid-derived LTE_4_ and PGF_2α_ accompanied by reduced pro-resolving EPA-derived LXA_5_ (**4** in Figure 1) (López-Vicario et al., 2020). The omega-6 arachidonic acid, biosynthesized from the conversion of LA through desaturation and elongation reactions, is also incorporated into phospholipids of mitochondrial membranes. Arachidonic acid is the major pro-inflammatory lipid mediator precursor and is able to replace the mitochondrial membrane LA leading to changes in membrane permeability, impaired function of respiratory chain complexes, higher cytochrome c release, ROS generation and mitochondrial depolarization through an uncoupling effect (Cocco et al., 1999; Scorrano et al., 2001; Haworth et al., 2010).

SPMs, firstly identified and termed by Serhan (2014), with well recognized potent inflammation-resolving effect on polymorphonuclear neutrophils and macrophages, are described to attenuate mitochondrial dysfunction in a mouse model of liver ischaemia/reperfusion injury and to reduce ROS levels in blood of patients with sepsis by increasing superoxide dismutase activity (Gu et al., 2018; Hecker et al., 2018; Kang et al., 2018). In the model of liver ischaemia/reperfusion injury, RvD1 which is biosynthesized from DHA *via* lipoxygenase (LOX) pathway (Serhan and Petasis, 2011), prevented ROS-mediated mitochondrial dysfunction and reduced mitochondrial swelling, lipid peroxidation and impaired activities of mitochondrial complexes I and III by regulating thioredoxin 2-mediated mitochondrial homeostasis (Kang et al., 2018). Moreover, RvD1 conserves ultrastructural morphology of mitochondria by activating mitophagy (Ren et al., 2020). In the sepsis model of bone marrow derived-macrophages (BMDM) with lipopolysaccharide (LPS), the DHA-derived 12-LOX product MaR1 (Serhan et al., 2009) was able to inhibit ROS production, increase mitochondrial membrane potential and improve ATP content (Gu et al., 2018).

Other lipid mediators worth mentioning are the EPA-derived SPM intermediate 18R-HEPE and RvE1, which also exerted a potent restorative effect in human peripheral mononuclear cells with TNFα-induced mitochondrial dysfunction (Hecker et al., 2018). After exposure to TNFα, cells treated with RvE1 and 18R-HEPE improved routine respiration and the mitochondrial membrane potential compared to those treated with the PUFAs arachidonic acid, DHA and EPA (Hecker et al., 2018). Interestingly, the impaired respiratory capacity and the impaired imbalance of mitochondrial fission and fusion were completely rescued by RvE1 (Mayer et al., 2019).

## 5 Mitochondrial Dysfunction at the Early Stages of Chronic Liver Injury

There is accumulating evidence that mitochondrial dysfunction contributes to the development and progression of fibrosis. Fibrosis development can be regarded as non-homeostatic innate immune response to tissue damage, and the role of hepatic stellate cells (HSCs) as innate immune cells is often under-recognized. Ongoing injury leads to activation of HSCs and their transdifferentiation into myofibroblasts, leading to progressive matrix deposition. This transformation is induced by inflammation-dependent and -independent mechanisms, including secretion of cytokines (i.e., TGFβ and IL-13), signaling through the succinate-GPR91-TGFβ pathway, mitochondrial ROS and apoptotic bodies arising from dying hepatocytes (Chiaramonte et al., 1999; Jiang et al., 2009; Pellicoro et al., 2014; Li et al., 2015a). Similar to immune cells, HSCs undergo metabolic rewiring during their activation. Hedgehog pathway is one of the main player in driving glycolysis and glutaminolysis in activated HSCs, which is essential for their transdifferentiation (Chen et al., 2012; Du et al., 2018). HSCs exert their immunomodulatory effects through production of ROS and pro-inflammatory cytokines and chemokines such as CC-chemokine receptor 2 (CCR2) ligand CCL2, CCL5 (RANTES), CCL3 and CCL4. Activated HSCs and macrophages secrete TGFβ, thereby further promoting myofibroblast activation and deposition of extracellular matrix (ECM). The canonical signaling of TGFβ is mediated by the Smad pathway, which results in repression of epithelial marker gene expression and activation of mesenchymal gene expression (Xu et al., 2009). However, it is less known that Smad2/3 are present and phosphorylated in mitochondria of CD4^+^ T cells, or that TGFβ impairs mitochondrial function by reducing the basal and ATP-coupled oxygen consumption rate (OCR) of human CD4^+^ T cells and inhibiting mitochondrial complex V (ATPase activity) (Dimeloe et al., 2019). Inhibition of complex V by TGFβ or oligomycin alone is sufficient to impair IFNγ production by CD4^+^ T cells. Therefore, through impairing mitochondrial respiration, TGFβ is able to induce T cell paralysis.

Interestingly, succinate also emerges as a key metabolite in the context of fibrosis development. Li et al. (2016) proposed that succinate functions as a paracrine signal between hepatocytes and HSCs, thereby inducing the succinate receptor GPR91 and upregulating the fibrogenic markers alpha-smooth muscle actin (α-SMA), TGFβ and collagen type I (**5** in Figure 1). As a proof of concept, inhibition of succinate-GPR91 signaling by Ly2405319, an analog of fibroblast growth factor (FGF)21, attenuated HSC activation.

Mitochondria do not only function as a platform for profibrogenic signaling, but also actively participate in fibrogenesis. Mitochondria-derived DAMPs (mtDAMPs) are particularly immunogenic due to their structural similarities to bacteria. mtDNA from hepatocytes released into circulation promote inflammation through binding to endosomal TLR9 of liver resident Kupffer cells (**6** in Figure 1) (Garcia-Martinez et al., 2016). Apart from their pro-inflammatory effect, a recent elegantly conducted study demonstrated that mtDNA is also able to directly activate HSCs and trigger pro-fibrogenic responses in mice (An et al., 2020). More importantly, patients with non-alcoholic steatohepatitis with advanced fibrosis stage have higher circulating levels of mtDNA, supporting the finding that mtDNA are able to drive hepatic fibrogenesis (An et al., 2020).

Non-alcoholic fatty liver disease is a vivid example of how systemic immune responses are regulated by metabolism, and that harmful local fibrosis of the liver can develop if these systemic metabolic disturbances remain unresolved. Therefore, metabolic alterations should not only be regarded as a consequence of immune responses, but they should be at least considered as a root for immune cell impairment.

## 6 Organ Dysfunction and Failure in Acute Decompensation Cirrhosis and Acute-on-Chronic Liver Failure: Role of Mitochondrial Dysfunction

Systemic inflammation in patients with AD cirrhosis and ACLF require energetically expensive immune responses in order to produce inflammatory mediators, respiratory burst, and positive and negative acute-phase proteins (such as albumin) (Clària et al., 2016; Moreau et al., 2020). As a consequence, a competition for energy with other maintenance programs, including organ function homeostasis, takes place (Ganeshan et al., 2019). In patients with AD and ACLF, glucose is preferentially used in extra-mitochondrial pathways in detriment of ATP production (Moreau et al., 2020), which directly triggers life-threatening organ failure(s), a recurrent feature present in diseases characterized by systemic inflammation (Carré and Singer, 2008; Van Wyngene et al., 2018). The impaired ATP production is especially devastating for the liver whose ATP synthesis rate, estimated at 30 mM/min in healthy volunteers using *in vivo* ^31^P magnetization transfer experiment, is extraordinarily high compared to human skeletal muscle for example (Schmid et al., 2008).

Hence, mitochondrial dysfunction is typically found as an important hallmark in chronic liver diseases including cirrhosis (Mansouri et al., 2018). An overview of the findings related to mitochondrial dysfunction in patients with cirrhosis and ACLF and in different animal models of chronic liver injury is provided in Tables 1, 2, respectively. In an attempt to compensate for decreased OXPHOS and maintain ATP production, hepatocytes of rats induced to cirrhosis with carbon tetrachloride (CCl_4_) undergo a metabolic shift from OXPHOS to glycolysis (Nishikawa et al., 2014). In addition, inflammatory cells release a large amount of oxidants and cytokines (Piano and Angeli, 2021), such as TNFα which imitates the effect of uncouplers in mitochondria, contributing to the decrease in ATP production (Kastl et al., 2014; Song et al., 2019).

**TABLE 1:** Overview of key findings related to mitochondrial dysfunction in patients with cirrhosis and ACLF.

**Key findings**		Mitochondrial function/process
**Cirrhosis**		
Increased numbers of mitochondria and swelling in circulating leukocytes of patients with AD cirrhosis (Zhang et al., 2021)		Mitochondrial structure
Systemic oxidative stress in patients with compensated and decompensated cirrhosis (Alcaraz-Quiles et al., 2018)		Oxidative stress
Decreased hepatic expression of COX1 and COX2 in cirrhotic patients (CHILD-Pugh A-C) (Nishikawa et al., 2014)		OXPHOS
Elevated serum mtDNA levels in patients with NASH-associated cirrhosis (An et al., 2020)		Mitochondrial DAMPs
**ACLF**		
Systemic oxidative stress in patients with ACLF, i.e. elevated levels of oxidized form of albumin (Oettl et al., 2008; Alcaraz-Quiles et al., 2018)		Oxidative stress
Increased extra-mitochondrial utilization of glucose in circulating leukocytes of patients with ACLF (Zhang et al., 2021)		Glucose metabolism
Elevated serum levels of hexanoyl- and tetradecenoylcarnitine (Moreau et al., 2020)		Mitochondrial β-oxidation
Downregulation of *PDP2* in circulating leukocytes of patients with ACLF (Zhang et al., 2021)		Glucose metabolism/TCA cycle
Decreased succinate utilization in leukocytes of patients with ACLF (Zhang et al., 2021)		TCA cycle

Bax, BCL2 associated X; BDL, bile duct ligation; CCl4, carbon tetrachloride; COX, cytochrome c oxidase; DDC, 3,5-diethoxycarbonyl-1,4-dihydrocollidine; DEN, diethylnitrosamine; D-gal: D-galactosamine; eNOS, endothelial nitric oxide synthase; i.p., intraperitoneal; i.v., intravenous; LPS, lipopolysaccharide; ND6, NADH dehydrogenase 6; p.o., per os; OCR, oxygen consumption rate; Ppargc-1α, peroxisome proliferator-activated receptor gamma coactivator 1-alpha; PDP2, pyruvate dehydrogenase phosphatase catalytic subunit 2; TAA, thioacetamide; Tfam, mitochondrial transcription factor A.

**TABLE 2:** Overview of different animal models of chronic liver injury, cirrhosis and ACLF and findings related to mitochondrial functionality.

Experimental model	Admin. route and induction time	Species	Key findings	Mitochondrial function/process
**Animal models of chronic liver injury**				
DDC	p.o. 8–10 weeks	Mouse	Reduced respiratory control ratio and complex II activity in hepatocytes (Nikam et al., 2013)	OXPHOS
			Reduction of hepatic ATP content (Nikam et al., 2013)	OXPHOS
DEN	i.p. 8 weeks	Mouse	Inhibition of complex I and complex IV in liver mitochondria (Santos et al., 2012)	OXPHOS
**Animal models of cirrhosis**				
CCl_4_	p.o. 12–14 weeks for compensated cirrhosis and 26–28 weeks for decompensated cirrhosis	Rat	Increased hepatocyte mitochondrial mass (citrate synthase activity) (Nishikawa et al., 2014) and mitochondrial swelling (spectrophotometry) (Natarajan et al., 2006)	Mitochondrial structure
			Increased mitochondrial superoxide levels in hepatocytes and HSC (Vilaseca et al., 2017)	Oxidative stress
			Reduced basal OCR and maximal mitochondrial respiration in hepatocytes from failing cirrhotic livers (Nishikawa et al., 2014)	OXPHOS
			Decreased expression of *COX1*, *COX2*, *ND6* (Nishikawa et al., 2014)	OXPHOS
			Increased dependency on glycolysis as energy source (Nishikawa et al., 2014)	Glucose metabolism/TCA cycle
			Downregulation of hepatic *PDP2* (Nishikawa et al., 2014)	
TAA	i.p. up to 5 months	Rat	Mitochondrial swelling (Natarajan et al., 2006)	Mitochondrial structure
			Increased malondialdehyde in liver mitochondria (Natarajan et al., 2006)	Oxidative stress
			Decreased respiratory control ratio (Natarajan et al., 2006)	OXPHOS
BDL	4 weeks	Rat	Decrease in mitochondrial membrane potential (Arduini et al., 2011)	Mitochondrial membrane potential
			Reduced hepatic expression of transcription factors regulating mitochondrial biogenesis (*Ppargc-1α*, *Tfam*) (Arduini et al., 2011)	Mitochondrial biogenesis
			Reduced hepatocyte activities of complex I, II and III (Krähenbühl et al., 1994)	OXPHOS
			Reduced fatty acid oxidation and impaired ketogenesis (Krähenbühl et al., 1994)	Mitochondrial β-oxidation
**Animal models of ACLF**				
Combination	CCl4: inhalation 15–16 weeks	Rat	Increased hepatic superoxide levels, decrease in hepatic eNOS activation (Tripathi et al., 2018)	Oxidative stress
CCl_4_ + LPS and BDL + LPS	BDL: 28 days			
	LPS: i.p. or i.v. (acute injection)			
Porcine serum + LPS + D-gal	Porcine serum i.p. 11 weeks	Rat	Increased hepatic expression of Bax and decreased expression of Bcl-2 (Li et al., 2017)	Apoptosis
	LPS: i.v. + D-gal: i.p. (acute injections)			

BDL, bile duct ligation; CCl_4_, carbon tetrachloride; COX, cytochrome c oxidase; DDC, 3,5-diethoxycarbonyl-1, 4-dihydrocollidine; DEN, diethylnitrosamine; eNOS, endothelial nitric oxide synthase; i.p., intraperitoneal; i.v., intravenous; ND6, NADH, dehydrogenase 6; p.o., per os; OCR, oxygen consumption rate; Ppargc-1α, peroxisome proliferator-activated receptor gamma coactivator 1-alpha; PDP2, pyruvate dehydrogenase phosphatase catalytic subunit 2; TAA, thioacetamide; Tfam, mitochondrial transcription factor A.

In hepatocytes of cirrhotic rats, the OCR as a proxy for the functionality of ETC is similar to that of control hepatocytes at early stages of cirrhosis, but was significantly reduced at the terminal stage (Nishikawa et al., 2014). The decreased OCR could be a consequence of prolonged exposure of hepatocytes to inflammatory cytokines (Stadler et al., 1992). However, another study in murine hepatocytes detected an increment of OCR after one hour of TNFα-induced damage (Kastl et al., 2014). These findings suggest that the time of exposure to inflammatory cytokines determines if mitochondrial respiration is increased as a first effort of mitochondria to supply enough energy from OXPHOS, or if it is decreased as a secondary damage of prolonged inflammation.

Another aspect of mitochondrial dysfunction of the OXPHOS system in liver cirrhosis is the enhanced production of ROS (Chan, 2006; Natarajan et al., 2006). Oxidative stress produced by mitochondria induces apoptosis in hepatocytes and might consequently lead to deposition of scar tissue, i.e., fibrosis and cirrhosis, and eventually decompensation of liver function (Paradies, 2014; Luangmonkong et al., 2018).

In addition to failure of liver function, kidney, brain, coagulatory and circulatory failure and the combinations thereof are also part of the ACLF syndrome (Moreau et al., 2013). The kidney is one of the most energy-demanding organs in the human body (Wang et al., 2010). During the course of cirrhosis there is a marked decrease of the production of energy by mitochondria from tubular epithelial cells that can be explained by decreased number of mitochondria, swelling of individual organelles and disrupted cristae (Gomez et al., 2014; Tran et al., 2011; Brooks et al., 2009). Additionally, ROS released by damaged mitochondria contributes to oxidative stress, thereby amplifying inflammation and eventually inducing cell death of kidney cells (**7** in Figure 1) (Emma et al., 2016).

Another aspect in patients with decompensated cirrhosis is hyperammonemia, which correlates with organ failures and mortality (Shalimar et al., 2019) and impairs neutrophil function (Shawcross et al., 2008), further establishing the intimate connection between disturbed mitochondrial intermediate metabolism and inflammation. Ammonium (NH_3_) is detoxified in mitochondria via glutamate or glutamine synthesis from α-KG, resulting in cataplerosis, i.e., depletion of the critical TCA cycle intermediate α-KG (Davuluri et al., 2016). Notably, in skeletal muscle this translates into sarcopenia due to lower flux of the TCA cycle and subsequent decreased ATP synthesis (**8** in Figure 1) (Jacobsen et al., 2001). Sarcopenia in turn results in reduced NH_3_ clearance from the circulation, as skeletal muscle contain significant amounts of glutamine synthetase, thereby favouring the development of hepatic encephalopathy (**9** in Figure 1) (Merli et al., 2013).

In the brain, the cells most vulnerable to ammonia toxicity are astrocytes (Norenberg, 1981). NH_3_ inhibits cerebral glutaminase activity (Bradford and Ward, 1976) and state III respiration in mitochondria isolated from acute NH_3_-intoxicated rats (Kosenko et al., 1997). It collapses the mitochondrial membrane potential and increases the mitochondrial permeability in cultured astrocytes, possibly through alkalinization and dissipation of the mitochondrial proton gradient. In sparse-fur mice, which are deficient in hepatic ornithine transcarbamylase and therefore congenitally hyperammonemic, a progressive inhibition of electron transport complexes, particularly complex IV (cytochrome c oxidase), was observed (Rama Rao et al., 1997). In addition, the production of glutamine, derived from NH_3_ detoxification, might similarly contribute to the induction of the mitochondrial permeability transition (MPT), since inhibition of glutamine synthetase with methionine sulfoximine (MSO) blocks the NH_3_-induced collapse of the mitochondrial membrane potential (Bai et al., 2001).

## 7 Therapeutic Strategies

In this part, we will majorly focus on therapeutic approaches targeted at reversal of the metabolic reprogramming that takes place in advanced liver cirrhosis and ACLF. For strategies based on reduction of oxidative stress levels in cirrhosis, we refer to the excellent review by Li et al. (2015b).

### 7.1 Targeting the Tricarboxylic Acid Cycle in Macrophages

Supporting anaplerotic reactions, for example by feeding glutamine into the TCA cycle, restores the phagocytic capacity of monocytes which were conditioned with plasma of ACLF patients (Korf et al., 2019). This can be achieved through inhibition of the glutamine synthetase by MSO, a sulfoximine derivative of methionine, or by directly feeding the TCA intermediate α-KG, which promotes M2 activation *via* Jumonji domain-containing protein D3-dependent epigenetic reprogramming (Liu et al., 2017). Another possibility to increase intracellular α-KG availability can be achieved by employing the cell-permeable analogue dimethyl-α-KG. Dimethyl-α-KG suppressed nuclear translocation of NF-κB in glutamine-deprived BMDMs. Furthermore, supplementation of α-KG can relieve nitro-oxidative stress via their antioxidant properties. α-KG treatment in HepG2 increased levels of L-carnitine and restored mitochondrial β-oxidation (Lemire et al., 2011). This is relevant in patients with AD, as they exhibit relative L-carnitine deficiency due to increased circulating acylcarnitines (Zhang et al., 2021). α-KG and succinate, both intermediates of the TCA cycle, act as antagonistic players, thus targeting the α-KG/succinate ratio might be a good manipulation point to tailor macrophage immune responses in patients with ACLF (Liu et al., 2017).

### 7.2 Inhibition of Succinate-GPR91 Signaling

Since the discovery of succinate as a specific activator of GPR91 in 2004 (He et al., 2004), the involvement of the succinate-GPR91 axis in a multitude of diseases is being gradually unravelled. Activation of GPR91, a plasma membrane receptor, leads to intracellular release of arachidonic acid and production of PGE_2_, development of tubulo-interstitial fibrosis in diabetic nephropathy and enhanced activation of HSCs in the liver (Correa et al., 2007; Robben et al., 2009; Peti-Peterdi, 2010). Small molecule antagonists of human GPR91 were firstly identified in 2011, among them two orally bioavailable compounds termed 5g and 7e, identified through a systematic structure-function high-throughput screening analysis (Bhuniya et al., 2011). Another high-affinity and highly selective human antagonist denoted NF-56-EJ40 was identified later in 2019 (Haffke et al., 2019). These compounds could serve as a promising starting point for preclinical studies investigating their effects in advanced liver cirrhosis.

### 7.3 Induction of PGC-1α

Activation of peroxisome proliferator-activated receptor γ coactivator 1α (PGC-1α), the master regulator of mitochondrial biogenesis, is a target worth considering. Interestingly, it has been discovered that PGC-1α, apart from its nuclear localization, is also found in mitochondria, where it forms a multiprotein complex with mitochondrial transcription factor A (TFAM) (Aquilano et al., 2010). To our knowledge, only two direct activators of PGC-1α, ZLN005 (2-[4-(1,1-dimethylethyl)phenyl]-1H-benzimidazole) and Mogroside VI B, a cucurbitane glucoside, are available. These compounds have been only investigated in preclinical models of ischaemia-induced neuronal injury and diabetic db/db mice, where they show beneficial effects (Zhang et al., 2013; Xu et al., 2018). All the other pharmacological compounds (metformin, bezafibrate, activators of AMP-activated protein kinase and sirtuins) are indirectly aimed at PGC-1α activation.

### 7.4 Restoring Lipid Homeostasis

RVX-208 (2-[4-(2-hydroxyethoxy)-3,5-dimethylphenyl]-5,7-dimethoxy-4/3H)-quinazo-linone) is a small molecule which displaces bromodomain and extraterminal (BET) domains from chromatin and increases transcription of APOA1 gene, thereby increasing APO1 and HDL levels in humans (McLure et al., 2013; Picaud et al., 2013). Several phase I and II clinical trials investigating BET inhibitors for its anti-proliferative effect in solid and hematologic malignancies are registered, and one phase III study is currently recruiting patients with myelofibrosis for the treatment with a BET inhibitor in combination with ruxolitinib, a janus kinase (JAK) inhibitor. Given the fact that inhibition of bromodomain-containing protein 4, another member of BET proteins, abrogated activation of HSCs and even reversed liver fibrosis in mice treated with CCl_4_ (Ding et al., 2015), BET inhibitors should be evaluated for the treatment of liver fibrosis. One other effect of BET inhibitors, which should be kept in mind, is that they have the potential to reactivate HIV from latency (Banerjee et al., 2012).

Another more general strategy aimed at restoring lipid homeostasis in patients with AD could be the activation of the liver X receptor (LXR). LXR promotes cholesterol efflux through ATP-binding cassette transporters ABCA1 and ABCG1 and induces the expression of genes involved in elongation and unsaturation of fatty acids. Activation of LXRα in the liver also induces lysophosphosphatidylcholine acyltransfase 3 (LPCAT3) which mediates the synthesis of phospholipid-containing PUFAs, thereby decreasing inflammatory responses (Li et al., 2013; Rong et al., 2013). The increase in PUFAs decreases transcriptional responses of NF-κB target genes through altered histone acetylation in their promoter regions (Li et al., 2013), and LPCs ameliorate cytokine secretion and enhance bacterial clearance (Elsbach and Levy, 1968; Yan et al., 2004). Therefore, activation of LXR could be beneficial in patients with AD and ACLF whose plasma is characterised by a decrease in the total PUFA pool (López-Vicario et al., 2020). However, activation of LXRs could be a double-edged sword, as LXRs are highly expressed in haematopoietic stem cells and myeloid progenitor cells, in which they decrease the proliferative responses to IL-3 and GM-CSF (Murphy et al., 2011). LXR also increase expression of MER proto-oncogene tyrosine kinase (MERTK), which suppresses TLR-4 mediated inflammatory responses through enhancement of efferocytosis. ACLF is on the one hand characterized by an exaggerated hyperinflammatory state, and on the other hand by immune paralysis as evidenced by an expansion of immunosuppressive acting monocytes and macrophages which express MERTK. In line with this, MERTK inhibitors restore cytokine production by immune cells from patients with ACLF (Bernsmeier et al., 2015). Therefore, because of the delicate balance between too much inflammation and not enough in patients with ACLF, a generalized therapy concept might be harmful for some patient subsets with ACLF. Therapy in patients with ACLF requires individualized concepts, and diagnostics should also intend to evaluate the extent of inflammation/immune dysfunction in each patient individually. In this case, an elegant solution as to how LXR activation can be targeted to specific cell subsets which exhibit a pro-inflammatory phenotype, but not to those which show exhaustion, would be welcome.

Last but not least, treatment of patients with cirrhosis should take dietary habits and physical activity, which are able to modulate mitochondrial function via direct or indirect mechanisms, into account. The analysis of the mitochondrial membrane phospholipidome in healthy subjects demonstrated that EPA and DHA supplementation is able to increase omega-3 PUFA levels in mitochondrial membranes from skeletal muscle biopsies by replacing omega-6 PUFA in mitochondrial phospholipids (Herbst et al., 2014). This study demonstrated the role for omega-3 PUFAs in the mitochondrial membrane reorganization while ameliorating mitochondrial function and ADP sensitivity (Herbst et al., 2014). Importantly, dietary omega-3 PUFA enrichment favored ATP-linked OCR in both peripheral mononuclear cells and macrophages (Lau et al., 2020).

## 8 Conclusion

The critical mass of research supports the importance of systemic inflammation in development of AD cirrhosis and ACLF. In this review, we focused on the emerging evidence that mitochondrial dysfunction is another hallmark of advanced liver cirrhosis. Mitochondria are not only the executers of the immune system, but they also act as powerful effectors endowed with the ability to shape the immune responses towards a hyperinflammatory or immunosuppressive state. Therefore, we suggest that future strategies to treat patients with AD cirrhosis and ACLF should be focused on restoring metabolic homeostasis in immune cells. In the Supplementary Material of this review, we summarize the methodological background of the current methods dedicated to the assessment of mitochondrial function.
