# Supplementary material for: Mitochondrial Dysfunction in Advanced Liver Disease: Emerging Concepts
Source: Front Mol Biosci. 2021 Nov 23;8:772174. doi: 10.3389/fmolb.2021.772174 (PMC8650317; doi:10.3389/fmolb.2021.772174)
Supplement: Supplementary file 1 [file DataSheet1.docx]

**Appendix**

1. *Assessment of whole cell flux analysis and mitochondrial function assays in leukocytes*

Background: Biolog’s PM-M1 and MitoPlate S-1 are assays based on the production of NADH and FADH_2_ from various substrates, which feed electrons to the ETC. A tetrazolium-based redox dye acts as an electron acceptor at the distal end of the ETC and the color formation, which indicates the utilization of the substrate present in each well, is read kinetically. PM-M1 plates can be used to phenotype multiple energy-producing pathways of the cells as previously described, such as glycolysis and pentose phosphate pathway.^1^ PM-M1 plates are provided as microplates in a 96-well format precoated with 91 carbon- and nitrogen sources in the low millimolar range (Biolog, Hayward, CA) (see **Table S1** for a detailed list). MitoPlate S-1 is suited to specifically phenotype the mitochondrial intermediate metabolism. It contains substrates exclusive to the tricarboxylic acid (TCA) cycle and amino acids in three technical replicates (see **Table S2** for a detailed list). Cytosolic substrates (D-glucose, glycogen, D-glucose 1-phosphate, D-glucose 6-phosphate, 6-phospho-D-gluconate, and lactate) are included in this assay and serve as negative controls in the MitoPlate S-1. Pyruvate and some mitochondrial substrates (L-leucine, α-aminobutyric acid, α-ketoisocaproic acid, acetylcarnitine, octanoylcarnitine and palmitoylcarnitine) are tested in the presence of sparker malate at 100 μM.

Procedure: For PM-M1 plates, the cells of interest are resuspended in IF-M1 medium supplemented with 5% FBS, penicillin/streptomycin and 0.3 mM glutamine followed by inoculation of 120000 (50 µl) leukocytes per well. After incubation for 24 h at 37°C in a 5% CO_2_ incubator, 10 µl of Redox Dye MB is added to each well and the kinetic reading should be immediately carried out in an OmniLog instrument (Biolog Inc., Hayward, CA) which uses a CCD camera to capture the purple color formation every 5 min at 37°C, for a time period of up to 24 h, depending on the metabolic activity of the cells. The background is corrected for the blank sample. The Biolog Data Analysis software can be used to calculate different parameters such as initial rate, plateau values or area under the curve.

The substrates on MitoPlate S-1 are first dissolved by incubating the plate with 30 µl of Assay Mix, consisting of 2x BMAS, 6x Redox Dye MC and saponin (30 µg/ml) necessary for cell permeabilization in a 5% CO_2_ incubator at 37°C for 1 h before inoculating 100000 leukocytes per well in a volume of 30 µl. Alternatively, when working with hepatocytes, cells are previously seeded in a 96-well plate (40000 per well) incubated during 3 hours for attachment and washed. The substrates dissolved in 30 µl of Assay Mix are then transferred to the 96-well plate containing the attached hepatocytes. The reading of the signals is immediately carried out as described above.

1. *Polarographic oxygen sensor and traditional oxygraphy*

Background: The polarographic oxygen electrode is the classic method for respirometry. Basically, the oxygen concentration is monitored in a closed chamber using a Clark electrode for the measurement of oxygen consumption by biological samples.^2^ The Clark electrode consists of a gold or platinum cathode and Ag/AgCl anode separated by a KCl solution. When voltage is applied, the dissolved O_2_ diffuses from the solution through an O_2_-permeable membrane (usually Teflon) and is reduced to water by electrons at the cathode, yielding hydroxide. The anode reaction is the oxidation of Ag precipitating at the Ag/AgCl anode yielding stream proportional to the O_2_ partial pressure which is converted into oxygen concentration. Importantly, the temperature is regulated and maintained constantly throughout the experiment.

The oxygraph plots the oxygen concentration over time yielding a slope, but artefacts and instrumental background noises cannot be discarded. Several issues can be found in traditional oxygraphy such as the signal instability due to irregular sensor behavior; the oxygen consumption of the sensor, although being smaller than respiration, is not quantified; the linearity of the slope is assumed whereas respiration capacity is nonlinear; limited time for titrations once oxygen is consumed; the oxygen back-diffusion could impede respiration measurements; and autoxidation of some substrates requires correction of respiration.

To date the analysis of mitochondrial function has relied on the classical oxygen electrode devices.^3^ The oxygen electrode method is inexpensive and a useful tool for the assessment of mitochondrial respiration. However, it has limitations as mentioned above. Considering the need for more accurate systems, new technologies have been generated such as the high-resolution Oxygraph-2k (O2k, Oroboros Instruments, Austria) and the sensitive high-throughput Seahorse XF Extracellular Flux Analyzer (Seahorse XF, Seahorse Bioscience Inc.).

- 1. *Oxygraph-2k, Oroboros*

Background: The O2K (Oxygraph-2k, Oroboros) is an *in vitro* assay device containing two separate 2 mL chambers equipped with polarographic oxygen sensors to monitor the oxygen concentration and calculate the OCR parameter. Substrates, uncouplers or inhibitors for mitochondrial activity are directly added into the injection ports of each chamber while sample suspensions (cells/isolated mitochondria) are continuously stirred. O2K integrates fluorometry and spectrophotometry technologies for the analysis of mitochondrial respiration and membrane potential.^4^

Procedure in permeabilized hepatocytes: Mitochondrial respiration is measured by high-resolution respirometry at 37ºC. Hepatocytes are seeded in a 6-well plate and after the corresponding stimuli, are trypsinized and resuspended in MiR05 respiration buffer (20 mM Taurine, 0.5 mM EGTA, 3 mM MgCl_2_-6H2O, 60 mM K-lactobionate, 10 mM KH_2_PO_4_, 20 mM HEPES, 110 mM D-sucrose, and 1 g/L fatty acid-free bovine serum albumin (BSA)). Cells are counted before adding 200 uL into the respiration chamber (final volume 2 mL). In order to assess the functionality of the ETC, ATP synthase inhibitor oligomycin (2 µM) is added to measure the respiration not related to ATP production. Next, mitochondrial uncoupler carbonyl cyanide *m*-chlorophenyl hydrazine (CCCP) (0.7 µM) is added until the maximum OCR is reached, and finally complex I inhibitor rotenone (1 µM) and complex III inhibitor antimyicin A (1 µM) are injected to measure the non-mitochondrial respiration.

Procedure in isolated mitochondria from liver tissue: 500 mg of liver tissue are homogenized using a polytetrafluorethylene pestle in ice-cold buffer (250 mM sucrose, 50 mM KCl, 5 mM EDTA, 5 mM MgCl_2_, 1 mM Na_4_P_2_O_7_, protease inhibitor (Complete Mini; Roche, pH 7.4) and centrifuged at 740*g* for 5 min at 4°C. Supernatants are centrifuged at 9000*g* for 15 min at 4°C. The pellet is resuspended in 1 mL of ice-cold buffer and centrifuged again at 10000*g* for 15 min at 4ºC. The final mitochondria-enriched pellet is resuspended in 500-1000 µl of ice-cold buffer and used for the measurement of oxygen consumption at 37ºC. 500 µg of the isolated mitochondria is brought to a final volume of 2.1 mL with respiration medium (0.5 mM EGTA, 3 mM MgCl_2_•6H_2_O, 20 mM taurine, 10 mM KH_2_PO_4_, 20 mM HEPES, 1 g/L BSA, 60 mM K-lactobionate, 110 mM sucrose, pH 7.1) and added into the oxygraph chamber. Resting respiration (state 4, absence of adenylates) is assessed by the addition of 10 mM glutamate and 2 mM malate as the complex I substrate supply and state 3 respiration is assessed by the addition of 2.5 mM ADP. Respiratory control ratio (RCR) is calculated as the quotient state 3/state 4. The integrity of the outer mitochondrial membrane is established by the addition of 10 μM cytochrome c. The addition of 10 mM succinate provides the state 3 respiration with parallel electron input to complexes I and II. ADP control of coupled respiration and uncoupling control are examined through the addition of the protonophore carbonylcyanide-4-(trifluoromethoxy)-phenylhydrazone (FCCP) (optimum concentration for maximal flux). The O_2_ flux within complex II substrate is assessed by inhibition of complex I with 0.5 μM rotenone. Non-mitochondrial respiration is assessed by inhibiting complex III with 2.5 μM antimycin A.

*2.2 Seahorse XF Extracellular Flux Analyzer: Mito Stress Test Kit*

Background: The Seahorse XF24 and XF96 extracellular flux analyzers use a piston to reversibly enclose a small volume (7 μl in the XF24) above the cells, monitoring oxygen uptake in that volume for up to 5 min, then raising the piston, allowing up to four injections of substrates and/or uncouplers through the ports during the experiment. When running the protocol of the Mito Stress Test Kit, three of the four ports are occupied by the inhibitors of the ETC, allowing only one additional injection, which is a limitation of this system.

Procedure: The following protocol is adapted to peripheral blood leukocytes and the Seahorse XFe24 Analyzer. The day prior to assay, the Seahorse cell culture microplates should be precoated in order to immobilize non-adherent cells during the assay. In case of human peripheral mononuclear cells, poly-L-lysine 0.01% solution can be used. After isolation, between 500000 and 700000 cells in a volume of 100 μl of assay medium (containing XF DMDM medium pH 7.4, 10 mM glucose, 1 mM sodium pyruvate and 2 mM L-glutamine) are seeded into each well. The Seahorse cell culture microplate is centrifuged at 200*g* (zero braking) for 1 min and transferred into a 37ºC incubator not supplemented with CO_2_ for 25 minutes. 400 μl of assay medium is added to each well, followed by another incubation at 37ºC without CO_2_ for 25 minutes before loading the Seahorse cell culture microplate into the Seahorse XFe24 Analyzer following the manufacturer’s Mito Stress Test protocol. For hydrating the sensor cartridge and loading the inhibitors into the ports (oligomyin, FCCP, rotenone/antimycin A), we refer to the Agilent Seahorse XF Cell Mito Stress Test Kit User Guide (Kit103015-100).

| **Poly- and oligosaccharides** | **Sugar derivative** | **Ketone bodies** |
| --- | --- | --- |
| Cyclodextrin | α-Methylglucoside | Acetoacetate |
| Dextrin | β-Methylglucoside | β-Hydroxybutyrate |
| Glycogen | Salicin | **Nucleosides** |
| Maltotriose | N-Acetylglucosamine | Thymidine |
| Mannan | α-Methylmannoside | Uridine |
| Melezitose | N-Acetylmannosamine | Adenosine |
| Stachyose | L-Arabinose | Inosine |
| Raffinose | D-Arabinose | **Amino acid metabolism** |
| Pectin | Lactulose | γ-Aminobutyric acid |
| **Disaccharides** | **Sugar alcohol** | α-Ketobutyrate |
| Maltose | Maltitol | α-Hydroxybutyrate |
| Trehalose | D-Sorbitol | γ-Hydroxybutyrate |
| Gentiobiose | D-Mannitol | **Fatty acids** |
| Sucrose | Adonitol | Butyric acid |
| Turanose | Xylitol | Propionic acid |
| Lactose | myo-Inositol | Acetic acid |
| **Monosaccharides** | Erythritol | Hexanoic acid |
| L-Glucose | Lactitol | **Alcohols** |
| D-Glucose | **Sugar acid** | 1,2-Propanediol |
| D-Mannose | D-Glucosaminic acid | Ethanolamine |
| D-Tagatose | D-Glucuronic acid | Glycerol |
| L-Sorbose | N-Acetylneuraminic acid | 2,3-Butanediol |
| L-Rhamnose | **Organic acid** | 3-Hydroxy-2-butanone |
| L-Fucose | Citrate | **G3P shuttle** |
| D-Fucose | Lactate | sn-Glycerol 3-phosphate |
| D-Fructose | Methylpyruvate | **Others** |
| D-Galactose | Pyruvate | D-Cellobiose |
| Sedoheptulosan | α-Ketoglutarate | 3-Methylglucose |
| **Monosaccharide ester** | Succinate | Palatinose |
| D-Glucose 6-phosphate | Monomethylsuccinate | Melibionic acid |
| D-Glucose 1-Phosphate | L-Malate | D-Melibiose |
| D-Fructose 6-phosphate | D-Malate | α-Methyl-D-galactoside |
|  | meso-Tartaric acid | β-Methyl-D-galactoside |
|  |  | β-Methyl-D-Xyloside |
|  |  | Methyl-D-lactate |
|  |  | Succinamic acid |
|  |  | Chondroitin sulfate C |
|  |  | Tricarballylic acid |

**Table S1:** Substrates tested in the whole cell flux assay (PM-M1).

**Table S2:** Substrates tested by the mitochondrial function assay (MitoPlate S-1).

| **TCA intermediates/substrates** | **Amino acid metabolism** | **Ketone body** |
| --- | --- | --- |
| Pyruvate* | α-Ketobutyrate | β-Hydroxybutyrate |
| Pyruvate | L-Glutamine | **Others** |
| Citrate | L-Glutamate | Sparker malate control |
| cis-Aconitate | L-Serine | D-Glucose |
| Isocitrate | L-Ornithine | Glycogen |
| α-Ketoglutarate | Tryptamine | D-Glucose 1-phosphate |
| Succinate | L-Leucine* | D-Glucose 6-phosphate |
| Fumarate | γ-Aminobutyric acid* | 6-Phospho-D-gluconate |
| Malate | α-Ketoisocaproic acid* | Lactate |
| **G3P shuttle** | Ala-Gln |  |
| sn-Glycerol 3-phosphate | **Fatty acid oxidation** |  |
|  | Acetylcarnitine* |  |
|  | Octanoylcarnitine* |  |
|  | Palmitoylcarnitine* |  |
|  |  |  |

* These substrates additionally contain malate in a concentration of 100 µM.

**References**

[1] Bochner BR, Siri M, Huang RH, Noble S, Lei XH, Clemons PA, et al. Assay of the multiple energy-producing pathways of mammalian cells. PLoS One 2011;6:1–8.

[2] Gnaiger E. Polarographic Oxygen Sensors , the Oxygraph , Respirometry To Assess. Drug-Induced Mitochondrial Dysfunct 2008:327–48.

[3] Chance B, Williams GR. Respiratory enzymes in oxidative phosphorylation. III. The steady state. J Biol Chem 1955;217:409–27.

[4] Gnaiger E. Mitochondrial Pathways and Respiratory Control An Introduction to OXPHOS Analysis. 2014.
